# Biochemical, Biophysical and Functional Characterization of an Insoluble Iron Containing Hepcidin–Ferritin Chimeric Monomer Assembled Together with Human Ferritin H/L Chains at Different Molar Ratios

**DOI:** 10.3390/cimb44010009

**Published:** 2021-12-28

**Authors:** Mohamed Boumaiza, Imene Fhoula, Fernando Carmona, Maura Poli, Michela Asperti, Alessandra Gianoncelli, Michela Bertuzzi, Paolo Arosio, Mohamed Nejib Marzouki

**Affiliations:** 1 Laboratoire d’Ingénierie des Protéines et des Molécules Bioactives, Institut Nationale des Sciences Appliquées et de Technologie BP 676, Tunis 1080, Tunisia; mnmarzouki@yahoo.fr; 2 Laboratoire Microorganismes et Biomolécules Actives, Faculté des Science de Tunis, Université de Tunis El Manar, Tunis 2092, Tunisia; fhoulaimene@gmail.com; 3 Molecular Biology Laboratory, Department of Molecular and Translational Medicine, University of Brescia, Viale Europa 11, 25123 Brescia, Italy; fernando.carmona.ra@gmail.com (F.C.); maura.poli@unibs.it (M.P.); michela.asperti@unibs.it (M.A.); 4 Proteomics Platform, Department of Molecular and Translational Medicine, University of Brescia, Viale Europa 11, 25123 Brescia, Italy; alessandra.gianoncelli@unibs.it (A.G.); michelabertuzzi29@gmail.com (M.B.)

**Keywords:** camel hepcidin, human hepcidin, ferroportin, human ferritin H/L chains, iron, mass spectroscopy, J774 cells, HepG2 cells

## Abstract

Hepcidin and ferritin are key proteins of iron homeostasis in mammals. In this study, we characterize a chimera by fusing camel hepcidin to a human ferritin H-chain to verify if it retained the properties of the two proteins. The construct (HepcH) is expressed in *E. coli* in an insoluble and iron-containing form. To characterize it, the product was incubated with ascorbic acid and TCEP to reduce and solubilize the iron, which was quantified with ferrozine. HepcH bound approximately five times more iron than the wild type human ferritin, due to the presence of the hepcidin moiety. To obtain a soluble and stable product, the chimera was denatured and renatured together with different amounts of L-ferritin of the H-chain in order to produce 24-shell heteropolymers with different subunit proportions. They were analyzed by denaturing and non-denaturing PAGE and by mass spectroscopy. At the 1:5 ratio of HepcH to H- or L-ferritin, a stable and soluble molecule was obtained. Its biological activity was verified by its ability to both bind specifically cell lines that express ferroportin and to promote ferroportin degradation. This chimeric molecule showed the ability to bind both mouse J774 macrophage cells, as well as human HepG2 cells, via the hepcidin–ferroportin axis. We conclude that the chimera retains the properties of both hepcidin and ferritin and might be exploited for drug delivery.

## 1. Introduction

Iron is an essential element for cells and organisms that functions as a cofactor in many proteins and enzymes for many biochemical processes, such as DNA synthesis, oxygen transfer and energy metabolism [1].

The cytosolic and ubiquitous human ferritin is composed of H- and L-chains of 182 and 174 amino acids, respectively, that are iron-regulated and co-assemble to form heteropolymers [2]. In mammals, these two subunits have little propensity to form homopolymers, and the formation of H/L heterodimers, in different proportions, is shown to be preferred, thus originating a large number of isoferritins (H24L0, H22L2, …, H0L24) with a tissue-specific distribution [3,4]. The subunits fold in a four-helical bundle that can pack into shells made of 24 subunits [2]. Any ferritin type containing the H chain with ferroxidase activity is able to react with ferrous (Fe^2+^) and induce its oxidation to ferric (Fe^3+^) and its mineralization inside the ferritin cavity. Up to 4000 Fe atoms per molecule can be stored, although, in vivo, the saturation is variable and lower, and iron can be released when needed by a specific autophagic mechanism named ferritinophagy, which involves binding to a molecule named NCOA4 and its delivery to phagolysosome for protein degradation [5]. In vitro, the ferritin iron is stable and does not exchange among molecules, whereas it can be released slowly by strong Fe(III) chelators, such as desferroxamine [6], or in the presence of reducing agents, including sulfides [7]. A similar reductive mechanism may also occur in vivo, and it was reported that various molecules, such as the neurotoxin 6-hydroxydopamine (6-OHDA), can reduce and release ferritin iron without protein degradation [8].

In mammals, the systemic iron absorption and recycling are regulated by hepcidin via its binding to the iron exporter ferroportin, its receptor, and inducing its internalization and degradation [9]. Recently, it has been reported that the carboxy terminus of hepcidin binds to a metal-binding site of ferroportin and that the presence of iron increases the hepcidin binding affinity by approximately 80-fold [10].

Here, we characterized both a chimera hepcidin-H ferritin (HepcH:FTH) produced in *E. coli* in an insoluble form and its denaturation/renaturation to produce various heteropolymeric shells with the L-chain, and we used mass spectrometry to distinguish between folded and unfolded heteropolymers assembled with different molar ratios. A proteomic profile was generated for each HepcH-FTH/FTL heteropolymer assembled with specific molar ratios.

## 2. Materials and Methods

### 2.1. HepcH Solubilisation

The insoluble hepcidin–ferritin monomer (HepcH) was first solubilized with urea at different concentrations; first, from 1 M to 10 M, and then with 6 M guanidine hydrochloride. All reactions employing HepcH and FTH monomers were made in 6 M guanidine hydrochloride, 20 mM TRIS, pH 7.4.

### 2.2. Iron Release from the Insoluble HepcH Monomer

HepcH construct, in 6 M guanidine, was subjected to iron removal with 2 different reductants—ascorbic acid and tris(2-carboxyethyl)phosphine (TCEP)—and the iron released was quantified by the ferrozine method [11]. Both reductants, the ascorbic acid and TCEP, were prepared freshly just before use and added to the reaction mixtures in a large excess: protein: reductant = 1:110. Ferrozine was prepared freshly and added to the reaction mixture just before the addition of reductant to discard the presence of free Fe(II) in the protein prior to reduction. Ferrozine was added in excess: protein: ferrozine = 1:30. The Fe(II) released was then quantified, thanks to the Fe(II)-Ferrozine complex formation, every hour, by UV/vis at 562 nm, until a plateau was reached.

### 2.3. Protein Thiol Assay

In parallel to Fe(II) release, thiol quantification with Ellman’s reagent was carried out [12]. Ellman’s reagent ((5,5-dithio-bis-(2-nitrobenzoic acid), DTNB) was prepared freshly and added to the reaction mixture just before the addition of reductant to discard the presence of thiols in the protein prior to reduction. DTNB was added in a large excess: protein: DTNB = 1:30. The reaction measures by UV/vis were taken every hour at 412 nm for SH-Ellman formation until a plateau was reached. Prior to the thiol quantification, Ellman’s reagent (DTNB) was tested if it gave a signal when it reacted with any of the reductants used in absence of protein. The zero was achieved on the blank. Therefore, a standard curve has been generated to determine the experimental sulfhydryl concentration present in the sample.

### 2.4. Assembly of HepcH-FTH and HepcH-FTL Polymers

In order to obtain stable HecpH-FTH and HepcH-FTL hetheropolymers, the HepcH monomer was assembled using different molar ratios of HepcH/FTH and HepcH/FTL [13].

### 2.5. SDS-PAGE

Proteins were analyzed on native and denaturing conditions, respectively, on 7% and 12% PAGE.

### 2.6. Mass Spectrometry Analysis

Matrix-assisted laser desorption/ionization (MALDI) time-of-flight (TOF)/TOF mass spectrometry (MS) was carried out using AB Sciex 5800 instrument [14].

### 2.7. Oxidation of Cysteines Present in the Hepcidin after Refolding with Native Ferritins

Cysteine oxidation to disulfide bridges was carried out using a glutathione redox system (GSH/GSSG), as described by Jordan et al. (2009) [15]. Briefly, the refolded ferritin samples containing the hepcidin construct were dialyzed for 48 h against 0.4 mM GSH/0.4 mM GSSG at pH 7.5 in cold room in order to slowly oxidize cysteine thiols to disulfides in parallel with the gradual removal of the TCEP in order to avoid the intermolecular disulfide bridges formation, thus enhancing the intramolecular S-S formation. After 48 h of dialysis, the sample was thoroughly dialyzed at room temperature against Tris 20 mM pH 7.4 to remove the GSG/GSSG, and the resulting oxidized S-S hepcidin-H-Ferritin samples were run on a native acrylamide 12% gel. Some precipitation is observed in the samples, probably due to the formation of large aggregates between different ferritins during the cysteines oxidation.

### 2.8. Cellular Studies

Human hepatocellular carcinoma HepG2 and mouse monocyte-macrophage J774 cell lines (Lombardy and Emilia Romagna Experimental Zootechnic Institute) were cultured as described by Poli et al. (2011) and Delaby et al. (2005), respectively [16,17]. Briefly, HepG2 cells were cultured in minimal essential medium (PAA) with 10% fetal bovine serum (PAA), 40 μg/mL gentamicin and 1 mM L-glutamine. On the other hand, J774 cells were grown in DMEM (PAA Laboratories GmbH), 10% endotoxin-free fetal bovine serum (Euroclone), 0.04 mg/mL gentamicin (Euroclone) and 2 mM L-glutamine (PAA Laboratories GmbH). Both cell lines were maintained at 37 °C under 5% CO_2_. Effects of HepcH-FTH heteropolymer (ratio 1:5) and synthetic human hepcidin (control) treatments were studied regarding ferroportin expression in HepG2 and J774 cells, respectively. Expression of FPN1 was studied in HepG2 cells treated with ferric ammonium citrate (FAC; 100 µM) or desferrioxamine mesylate (DFO; 100 µM) for 16 h, and with the chimeric protein for 8 h. Cell lysate proteins (30 µg per line) were separated onto SDS-PAGE, electro-transferred onto nitrocellulose and analyzed with anti-ferroportin and anti-actin serum.

## 3. Results and Discussion

Hepcidin and ferritin are iron modulator molecules involved in iron homeostasis and iron storage, respectively [18]. Here, we demonstrated that, after fusing the two proteins, a bifunctional polymer is obtained with enhanced potential as an iron storage molecule, as well as an iron homeostasis regulator through ferroportin degradation in HepG2 and J774 cell lines.

### 3.1. Iron Removal Studies

**Figure 1:**
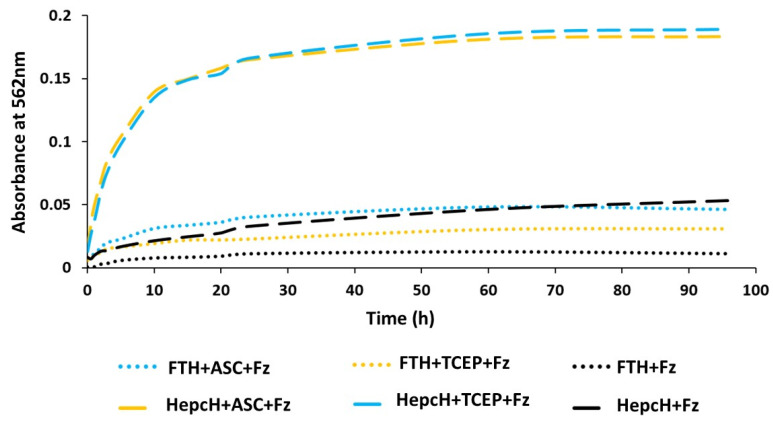
Iron release from HepcH subunit. This latter was incubated with the ascorbic acid (hepcH + ASC + Fz) and TCEP (hepcH + TCEP + Fz) reluctants or without (hepcH + Fz) in presence of ferrozine, which readily chelates iron with the formation of the Fe (II)-ferrozine complex that absorbs at 562 nm. Time course of iron release was performed till 96 h and a plateau was shown after 24 h of reaction. The recombinant human FTH was introduces in the analysis, as control. Results are presented as the mean of two independent experiments.

Our previous work has already demonstrated that the hybrid hepcidin–ferritin–H construct (HepcH) expressed in *E. coli* was properly purified as an iron-containing protein [13]. This was shown by its amber color and by the release of Fe(II) under reluctant conditions that formed a colored complex with ferrozine. In this study, we analyzed the Fe(II) release from HepcH and FTH insoluble preparations with two different reductants: ascorbic acid and TCEP in the presence of the chelator ferrozine that makes a colored complex that absorbs at 562 nm. The results showed that the HepcH construct releases Fe(II) in the presence of both the reductants tested, and that the amount of iron released was around five times bigger than that released by the sole human ferritin FTH (Figure 1; Table 1). In the absence of reducing agents, no iron was detected (Figure 1, black dashed and dotted lines), testifying that only the Fe(III) was bound to the HepcH monomer. The iron released after treatment with ascorbic acid and TCEP reached a concentration of 6.5 and 6.8 µM, respectively, corresponding to approximately 1.4 Fe atoms per HepcH monomer (Table 1). On the other hand, only approximately 1 µM of iron was detected after the treatment of the native FTH monomer with both reluctant agents, showing a negligible absorbance of the complex at 562 nm (Figure 1, turquoise and yellow dotted lines). The kinetics of the Fe(II) release from HepcH reached a plateau only after 24–30 h with both reductants (Figure 1), suggesting that the iron is bound to the protein moiety and not as a free form. The amount of iron Fe(II) detected in the HepcH construct after reduction with ascorbic acid or TCEP was at least five times higher than that of the native human ferritin (FTH), and, together with the slow kinetics of iron released, it suggested that the Fe(II) could form iron–sulfur clusters with the SH present in the hepcidin structure [19].

### 3.2. Quantification of Free Sulfhydryl Groups

**Figure 2:**
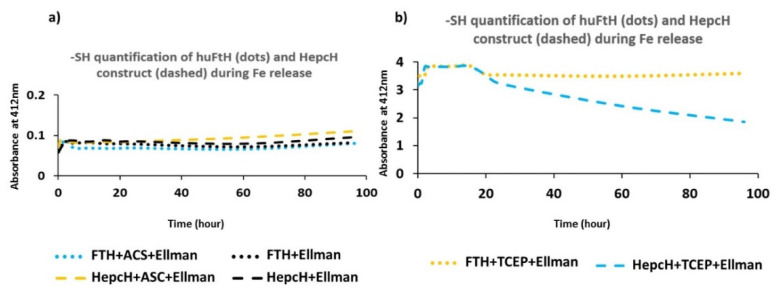
Time course of Ellman’s assay of FTH and HepcH during iron release. (**a**) Time course of Ellman’s assay of FTH and HepcH in presence of ascorbic acid reductant showing the absence of absorption bands at 412 nm. Turquoise dots: FTH reduced with ascorbic acid (FTH + ASC + Ellman). Black dots: non-reduced FTH (FTH + Ellman, control). Yellow dashes: HepcH reduced with ascorbic acid (hepcH + ASC + Ellman). Black dashes: non-reduced HepcH (hepcH + Ellman, control). (**b**) Time course of Ellman’s assay of FTH and HepcH in presence of TCEP reductant showing the presence of absorption bands at 412 nm. Yellow dots: FTH reduced with TCEP (FTH + TCEP + Ellman). Turquoise dashes: HepcH reduced with TCEP (hepcH + TCEP + Ellman). FTH and HepcH subunits reduced in presence of TCEP showed the presence of absorption bands at 412 nm with a pic after 18 h of incubation. Results are presented as the mean of two independent experiments.

Thiol quantification was performed by an absorbance measurement of purified HepcH and FTH monomers incubated with DTNB before and after reduction with ascorbic acid or TCEP. The results showed that the ascorbic acid did not reduce the HepcH and FTH thiol groups. This could be explained by the auto-oxidation of the ascorbic acid, which is significantly enhanced with the addition of increasing concentrations of iron [20]. In contrast, the TCEP efficiently reduced both protein thiols (Figure 2a versus Figure 2b). The kinetics of the reduction of HepcH and FTH monomers by the ascorbic acid and TCEP was monitored for 96 h (Figures S1 and S2), and a maximum absorbance of the cysteine rich molecule HepcH was observed after 15 h of post-reduction with TCEP (Figure S2e).

### 3.3. Assembly of HepcH-FTH/FTL with Different Molar Ratios

**Figure 3:**
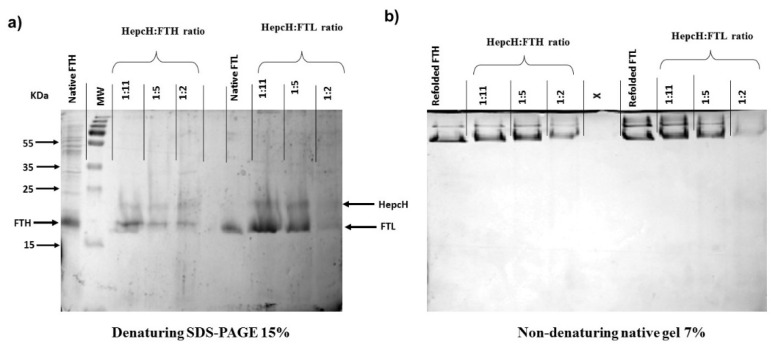
Assembly of HepcD-FTH and -FTL heteropolymers. (**a**) Denaturing SDS-PAGE analysis of the assembled HepcH-FTH and -FTL heteropolymers obtained after mixing different HepcH: FTH/FTL molar ratios (1:11; 1:5; 1:2). (**b**) Non-denaturing PAGE analysis of the assembled HepcH-FTH and -FTL heteropolymers at different ratios (1:11; 1:5; 1:2). Ratio HepcH:FTH or FTL = 1:1, corresponds to average number of 12 HepcH per 24 subunits. Ratio HepcH:FTH or FTL = 1:2, corresponds to average number of 8 HepcH per 24 subunits. Ratio HepcH:FTH or FTL = 1:5, corresponds to average number of 4 HepcH per 24 subunits. Ratio HepcH/FTH or FTL = 1:11, corresponds to average number of 2 HepcH per 24 subunits.

The HepcH insoluble subunit was denatured and then renatured together with wild type H- and L-ferritin chains with a molar ratio HepcH:FT of 1:5 [13]. Iron removal and the refolding experiments were carried out under reducing conditions. The presence of hepcidin at the N-terminus of the HepcH monomer inhibited its spontaneous assembly in a ferritin 24-mer shell. However, as previously shown, it was able to co-renature together with native human H- and L-ferritin chains at different molar ratios, such as HepcH:FT of 1:11, 1:5, 1:2, 1:1 and 5:1, producing heteropolymeric ferritin shells [13]. Heteropolymers were assembled at molar ratios of 1:11, 1:5 and 1:2, and were then analyzed under denaturing conditions on 12% SDS-PAGE, and under non-denaturing conditions on 7% native-PAGE (Figure 3a,b). The HepcH-FTH/FTL reassembly performed with an excess of the hepcidin–ferritin monomer (HepcH:FT of 1:2) was not efficient, and the products had the tendency to precipitate (Figure 3a,b). However, the reassembly with lower ratios of HepcH:FT of 1:5 and 1:11 showed the ability to produce a protein that was more stable and correctly refolded after the renaturation procedure (see Materials and Methods section). The results show that an efficient reassembly to a stable 24-mer shell containing the HepcH, both with FTH or FTL, occurs only under few HepcH to Ft ratios, and specifically those containing two, four or eight HepcH subunits per shell.

### 3.4. Mass Spectrometry Analysis of the Assembled Polymers

**Figure 4:**
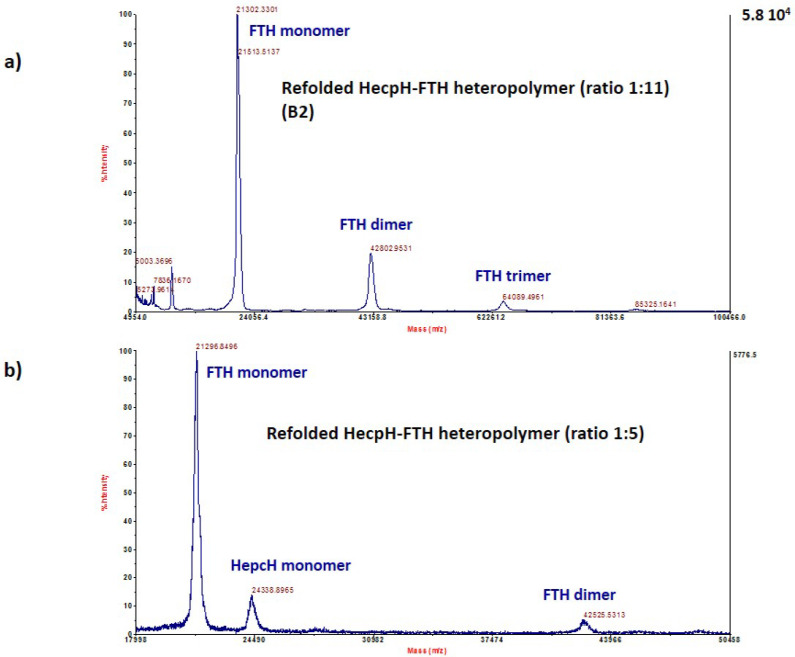
High-resolution MALDI-TOF mass analysis of the refolded HepcH-FTH heteropolymer. (**a**) MALDI-TOF mass analysis of the refolded HepcH-FTH heteropolymer, assembled using a molar ratio HepcH:FTH of 1:11, showing masses corresponding to FTH monomers, dimer and trimer. (**b**) MALDI-TOF mass analysis of the refolded HepcH-FTH heteropolymer, assembled using a molar ratio HepcH:FTH of 1:5, showing masses corresponding to HepcH and FTH monomers and FTH dimer.

**Figure 5:**
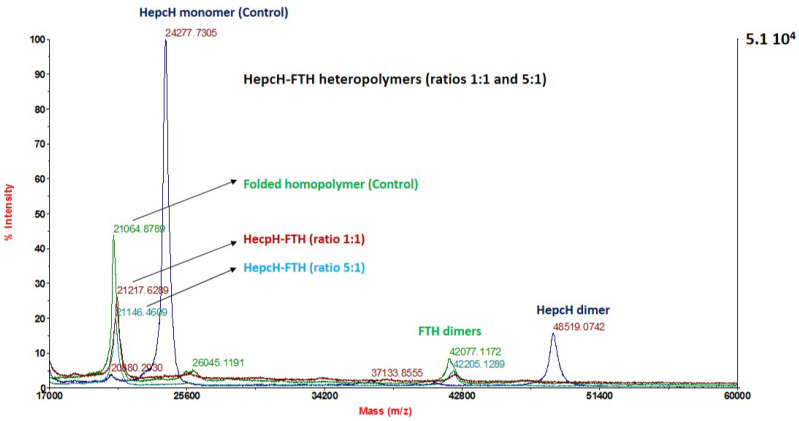
High-resolution MALDI-TOF/TOF mass analysis of the refolded heteropolymer assembled with high HepcH:FTH molar ratio. Folded human ferritin homopolymer (in green). HepcH-FTH heteropolymer, assembled using a molar ratio HepcH:FTH of 1:11 (in red). HepcH-FTH heteropolymer, assembled using a molar ratio HepcH:FTH of 5:1 (in turquoise). HepcH monomer (in dark blue).

**Figure 6:**
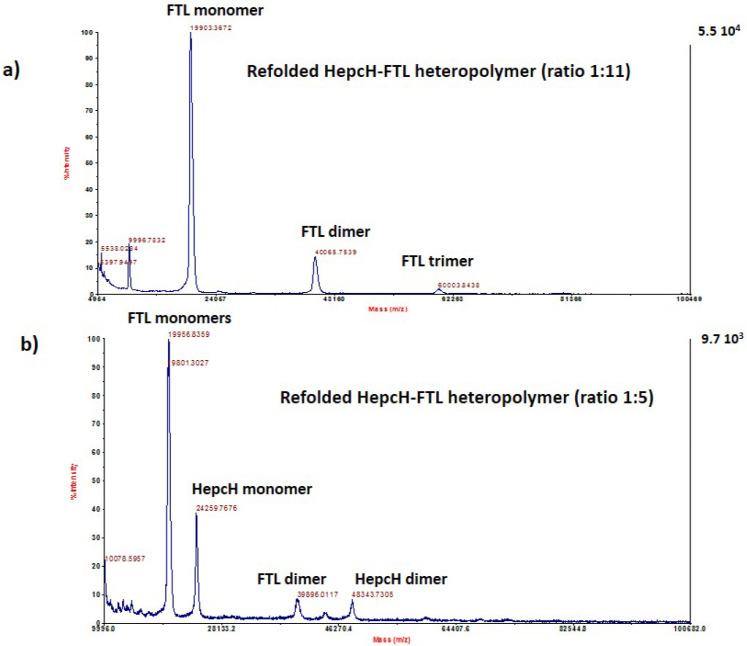
High-resolution MALDI-TOF/TOF mass analysis of the refolded heteropolymer HepcH-FTL. (**a**) MALDI-TOF mass analysis of the refolded HepcH-FTL heteropolymer, assembled using a molar ratio HepcH:FTL of 1:11, showing masses corresponding to FTL monomer, dimer and trimer. (**b**) MALDI-TOF mass analysis of the refolded HepcH-FTL heteropolymer, assembled using a molar ratio HepcH:FTL of 1:5, showing masses corresponding to HepcH and FTL monomers, FTL dimer and HepcH dimer.

We used MALDI-TOF/TOF-based analysis to characterize the different heteropolymers generated by HepcH assembling with different molar ratios of native H- and L-ferritin chains. This method was efficient in detecting both HepcH and FTH/FTL subunits of the assembled polymers. The analysis of the refolded ferritin with a molar ratio HepcH:FT of 1:5, corresponding to four HepcH per 24 subunits, detected peptides with masses of 21,296.8496 and 24,338.8965 Da, corresponding, respectively, to the FTH and HepcH monomers (Figure 4b. The heteropolymers with higher amounts of HepcH with molar ratios of HepcH:FTH of 1:1 and 5:1, corresponding to 12 and 20 HepcH monomers per 24 subunits, were also analyzed by mass spectroscopy. The results showed that only FTH subunits were detected, with experimental masses of 21,217.6289 and 21,146.4609, which correspond to the theoretical mass of the FTH monomer (Figure 5). On the other hand, the refolded heteropolymer with a molar ratio of HepcH: FT of 1:11, corresponding to two HepcH per 24 subunits, showed that it was composed of masses of 21,302.3301 and 19,903.3672, corresponding to FTH and FTL (Figure 4a and Figure 6a). We found that the HepcH monomer was detected only when mixed with FTL with a molar ratio of 1:1 (Figure S6). Native homopolymers of FTH and FTL were also analyzed by mass spectroscopy, giving components corresponding to the subunit monomer, dimer, trimer and tetramer (Figures S4 and S5). Thus, mass spectrometry proved to be an efficient method to identify the most stable heteropolymer assembled with the molar ratio HepcH:FT of 1:5, by detecting both HepcH and FTH/L subunits constituting the hybrid molecule.

### 3.5. Functional Assays of Free and Ferritin Exposed Hepcidins

**Figure 7:**
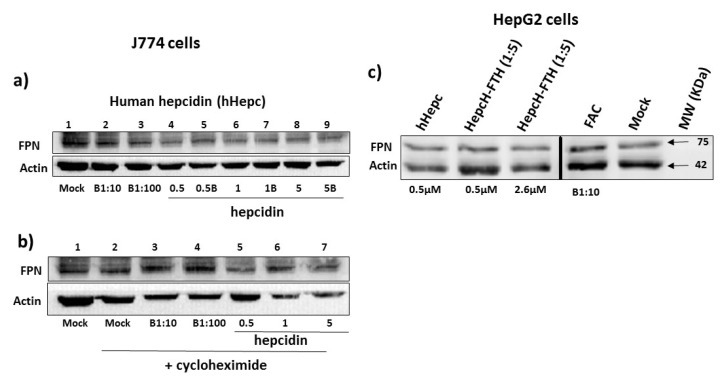
HepcH-FTH (1:5) promotes ferroportin degradation in HepG2 cells treated with iron. (**a**) Western blotting analysis of the denaturing 12%SDS-PAGE of J774 cell lysates after treatment with 0.5 μM to 5 µM human hepcidin (lane 4 to lane 9) during 2 h incubation using polyclonal anti-rabbit ferroportin antibody. Lane 1, mock (control). Lane 2, cells treated with 100 µM FAC for 16 h to enhance ferroportin expression. Lane 3, cells treated with 10 µM FAC. Lane 4, cells treated with 0.5 µM hepcidin (cells none treated with FAC). Lane 5, cells treated with *0.5 µM hepcidin* (cells treated with 100 µM FAC). Lane 6, *cells treated with 1 µM hepcidin* (cells none treated with FAC). Lane 7, cells treated with *1 µM hepcidin* (cells treated with 100 µM FAC). Lane 8, *cells treated with 5 µM hepcidin* (cells not treated with FAC). Lane 9, cells treated with *5 µM hepcidin* (cells treated with 100 µM FAC). (**b**) Western blotting analysis of the denaturing PAGE of J774 cell lysates after treatment with 0.5 μM to 5 µM human hepcidin during 2 h incubation, in the presence of cycloheximide (1 μg/mL), using polyclonal anti-rabbit ferroportin antibody. Lane 1, *mock cells (control). Lane 2, cells treated with 1 µg/mL* cycloheximide. Lane 3, cells treated with *1 µg/mL* cycloheximide and 100 µM FAC. Lane 4, cells treated with *1 µg/mL* cycloheximide and 10 µM FAC. Lane 5, cells treated with *1 µg/mL* cycloheximide and 0.5 µM hepcidin. Lane 6, cells treated with *1 µg/mL* cycloheximide and 1 µM hepcidin. Lane 7, cells treated with *1 µg/mL* cycloheximide and 5 µM hepcidin. (**c**) Western blotting analysis of the denaturing PAGE of cell lysates after treatment with 0.5 μM and 2.6 μM HepcH-FTH (1:5 and 1:11) during 2 h incubation using polyclonal anti-rabbit ferroportin antibody.

A mass spectroscopy analysis confirmed the presence of HepcH in the stable 24-mer shells assembled with FTH or FTL subunits with a molar ratio HepcH:FT of 1:5. Once refolded, cysteines present in the hepcidin construct (HepcH) were oxidized to disulfide S-S bridges, which are essential for the folding of biologically functional hepcidin on the ferritin surface. Two different hepcidin forms were analyzed for their biological activity toward ferroportin under different conditions. The mouse J774 human HepG2 cells were initially treated with ferric ammonium citrate (FAC; 100 µM) or desferrioxamine mesylate (DFO; 100 µM) to modulate the expression of ferroportin. In addition, J774 cells were also treated with cycloheximide (which blocks new protein expression), and then with different concentrations of synthetic human hepcidin (0.5 µM, 1 µM and 5 µM) in the presence or absence of 100 µM FAC. Immunoblotting showed that ferroportin was partially degraded in comparison to the non-treated cells (Figure 7a, lane 4 to 9 versus lane 1 to 3). When treated with FAC, ferroportin was more expressed than the untreated cells (Figure 7b, lane 1 and 2 versus lane 3 and 4). Furthermore, cycloheximide did not affect ferroportin expression in J744 cells (Figure 7b lane 1 versus lane 2) or the biological activity of human hepcidin toward ferroportin (Figure 7b, lane 2 to 4 versus lane 6 to 7). Ferroportin expression was increased in HepG2 cells treated with ferric ammonium citrate (FAC; 100 µM) (Figure 7c, Mock versus FAC). Furthermore, HepG2 cells treated with the 0.5 and 2.6 µM HepcH-FTH heteropolymer showed a decrease in their FPN protein level in a dose-dependent manner, compared to the untreated cells (Figure 7c, lines HepcH-FTH versus lines Mock and FAC). In HepcG2 cells, HepcH-FTH was effective towards FPN as human hepcidin, which was used as a control (Figure 7c, line hHepc). The correctly folded heteropolymer (1:5) with exposed camel hepcidin was also added to the HepG2 cells at concentrations of 0.5 µM and 2.6 µM, proving to also have the potential to bind and degrade the human FPN with the same efficacy as the human hepcidin (Figure 7, lane hHepc vs. lanes HepcH-FTH). Figure 7 shows that the treatment with cycloheximide, a protein synthesis inhibitor, did not inhibit human hepcidin activity toward FPN in J774 cells [21]. In this study, we characterized the bifunctional role of the HepcH-FTH heteropolymer as an iron homeostasis regulator and iron carrier. It could also be considered as an iron-chelating agent that can be useful for the clinical treatment of iron-overload syndromes, as well as in the treatment of other diseases characterized by oxidative stress, including cardiovascular disease, atherosclerosis, neurodegenerative diseases and cancer [22].

## Figures and Tables

**Table 1:** Iron concentration of HepcH and FTH monomers after treatment with ascorbic acid and TCEP.

Monomer	Reductant	Maximum Absorbance at 562 nm (after 60 h)	Fe(II) Concentration (µM)
FTH (9.4 µM)	Ascorbic acid	0.048 ± 0.01	1.7 ± 0.10
	TCEP	0.03 ± 0.00	1.1 ± 0.13
HepcH (9.4 µM)	Ascorbic acid	0.182 ± 0.03	6.5 ± 0.50
	TCEP	0.19 ± 0.05	6.8 ± 0.45

## Data Availability

The data presented in this study are contained within the article.

## Supplementary Materials

The following supporting information can be downloaded at: https://www.mdpi.com/article/10.3390/cimb44010009/s1, Figure S1: UV–vis spectra of Ellman’s assay of the oxidized and reduced forms of FTH subunit; Figure S2: UV–vis spectra of Ellman’s assay of the oxidized and reduced forms of HepcH hybrid subunit; Figure S3: High-resolution MALDI-TOF mass analysis of the FTH homopolymer; Figure S4: High-resolution MALDI-TOF mass analysis of the FTL homopolymer; Figure S5: High-resolution MALDI-TOF mass analysis of the HepcH-FTH heteropolymer, assembled using a molar ratio HepcH:FTL of 1:1.

## Abbreviations

FAC	Ferric ammonium citrate
HepcH	Camel hepcidin–human ferritin H fusion protein monomer
HepcH-FTH	Camel hepcidin–human ferritin H heteropolymer (assembled protein)
