# Supplementary material for: Biochemical, Biophysical and Functional Characterization of an Insoluble Iron Containing Hepcidin–Ferritin Chimeric Monomer Assembled Together with Human Ferritin H/L Chains at Different Molar Ratios
Source: Curr Issues Mol Biol. 2021 Dec 28;44(1):117–27. doi: 10.3390/cimb44010009 (PMC8929011; doi:10.3390/cimb44010009)
Supplement: Supplementary file 1 [file cimb-44-00009-s001.zip › cimb-1488387-supplementary.pdf]

Supplementary data

Figure S1

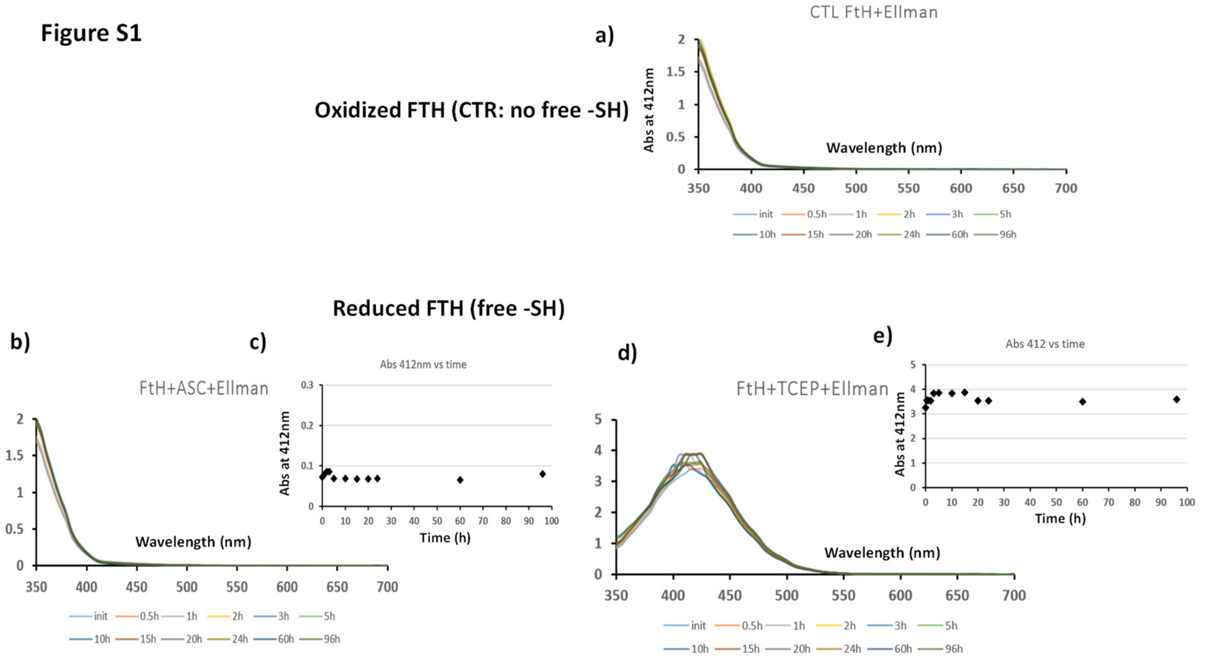

Figure S1 UV–vis spectra of Ellman's assay of the oxidized and reduced forms of FTH subunit. (a) UV–vis spectra of Ellman's assay of the oxidized FTH (control). UV–vis spectra (b) and time course (c) of Ellman's assay of FTH in presence of ascorbic acid reductant showing the absence of absorption bands at 412 nm. UV–vis spectra (d) and time course (e) of Ellman's assay of FTH in presence of TCEP reductant showing the presence of absorption bands at 412 nm.

Figure S2

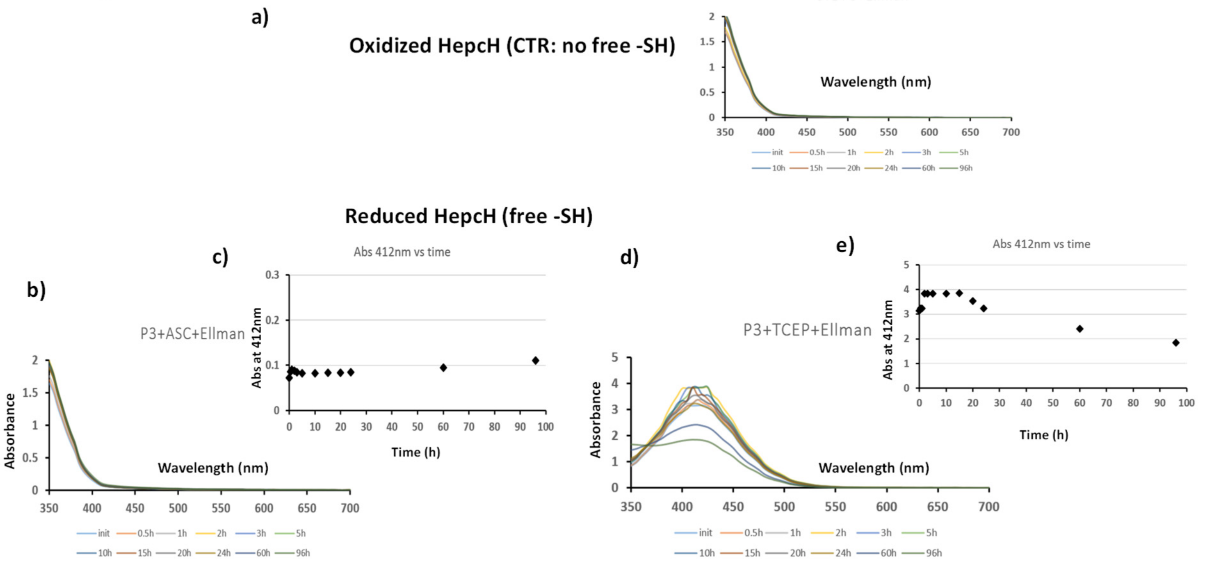

Figure S2 UV–vis spectra of Ellman's assay of the oxidized and reduced forms of HepcH hybrid subunit. (a) UV–vis spectra of Ellman's assay of the oxidized HepcH (control). UV–vis spectra (b) and time course (c) of Ellman's assay of HepcH in presence of ascorbic acid reductant showing the absence of absorption bands at 412 nm. UV–vis spectra (d) and time course (e) of Ellman's assay of HepcH in presence of TCEP reductant showing the presence of absorption bands at 412 nm.

Figure S3

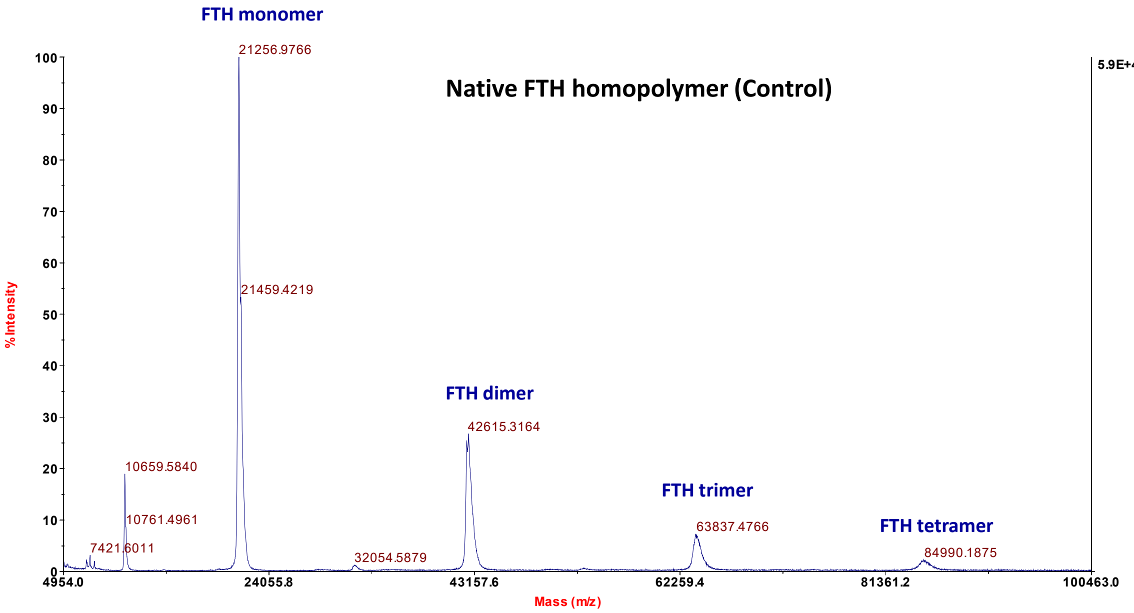

Figure S3 High-resolution MALDI-TOF mass analysis of the FTH homopolymer. Different peaks corresponding to masses of FTH monomers, dimer, trimer and tetramer.

Figure S4

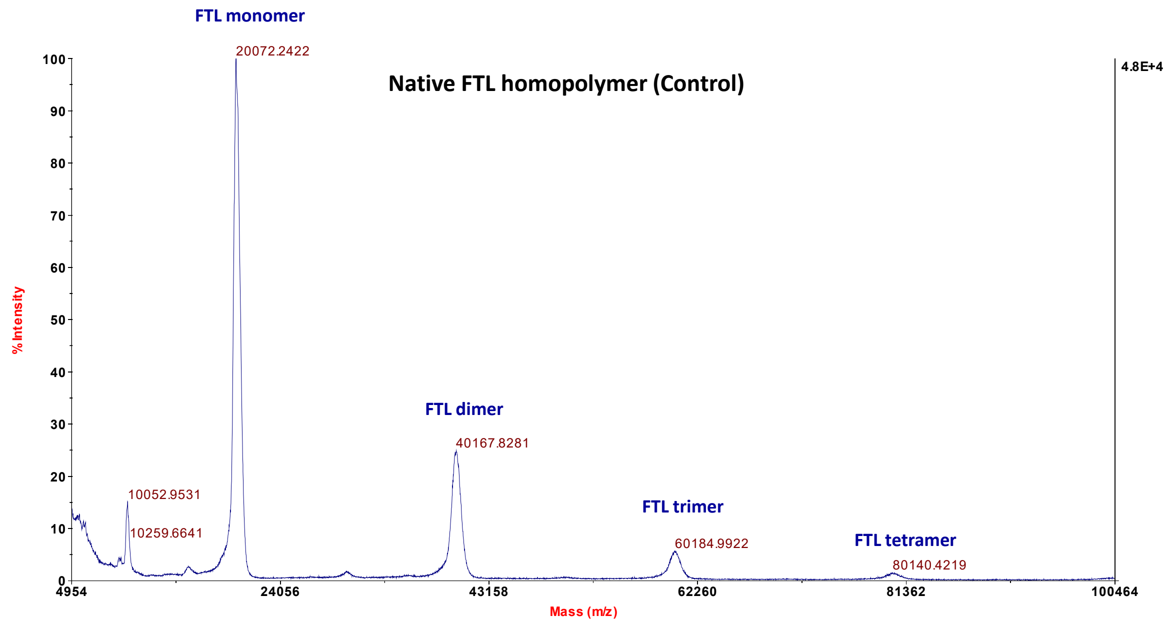

Figure S4 High-resolution MALDI-TOF mass analysis of the FTL homopolymer. Different peaks corresponding to masses of FTL monomer, dimer, trimer and tetramer.

Figure S5

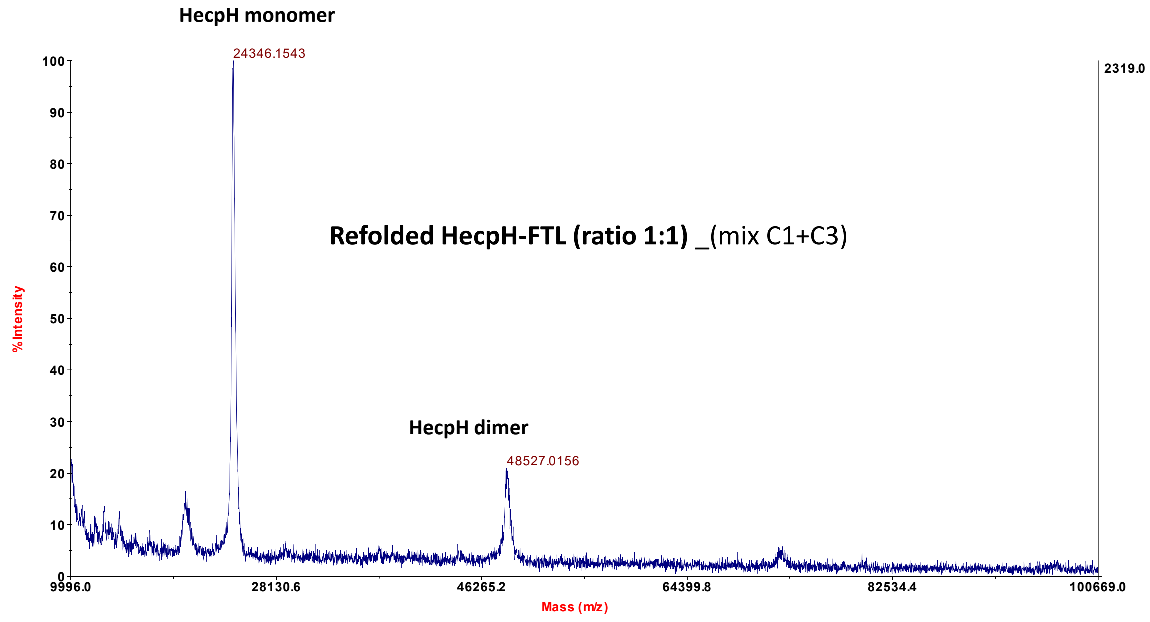

Figure S5 High-resolution MALDI-TOF mass analysis of the HepcH-FTH heteropolymer, assembled using a molar ratio HepcH:FTL of 1:1. Only peaks corresponding to masses of HepcH monomer and dimer was detected.
